# Rustrela Virus-Associated Encephalomyelitis (‘Staggering Disease’) in Cats from Eastern Austria, 1994–2016

**DOI:** 10.3390/v15081621

**Published:** 2023-07-25

**Authors:** Viktoria Weiss, Pia Weidinger, Julia Matt, Christiane Weissenbacher-Lang, Norbert Nowotny, Herbert Weissenböck

**Affiliations:** 1 Institute of Pathology, University of Veterinary Medicine, 1210 Vienna, Austria; 01051128@students.vetmeduni.ac.at (V.W.); julia.matt@vetmeduni.ac.at (J.M.); christiane.weissenbacher-lang@vetmeduni.ac.at (C.W.-L.); 2 Viral Zoonoses, Emerging and Vector-Borne Infections Group, Institute of Virology, University of Veterinary Medicine, 1210 Vienna, Austria; pia.weidinger@vetmeduni.ac.at (P.W.); norbert.nowotny@vetmeduni.ac.at (N.N.); 3 Department of Basic Medical Sciences, College of Medicine, Mohammed Bin Rashid University of Medicine and Health Sciences, Dubai P.O. Box 505055, United Arab Emirates

**Keywords:** cat, rustrela virus, encephalomyelitis, staggering disease, Austria

## Abstract

Clinical cases of ‘staggering disease’, a nonsuppurative encephalomyelitis associated with gait abnormalities in cats, have been documented for decades in Sweden. In Austria, an increased incidence was observed in the 1990s. Only recently, rustrela virus (RusV) was identified as the causative agent of this clinicopathologic disease entity. In this retrospective study, we analyzed a total of 23 brain and spinal cord samples from Austrian cats with the pathohistological diagnosis of nonsuppurative encephalomyelitis and clinical signs consistent with staggering disease from 1994 to 2016 using reverse transcription real-time polymerase chain reaction (RT-qPCR) and in situ hybridization. We were able to detect RusV nucleic acids in seven of the examined samples. Borna disease virus 1 (BoDV-1) could be excluded in all cases via immunohistochemistry and RT-qPCR. This study confirms that RusV has been a relevant etiological agent of nonsuppurative encephalomyelitis of cats in a geographically and temporally limited disease cluster in Austria, mainly in the 1990s. The geographic distribution of the positive samples in this study is consistent with earlier reports on ‘staggering disease’ in Austria. Further studies are necessary to confirm the reservoir host of ‘staggering disease’ in Austria, as well as investigations on the disappearance of this disease and its possible zoonotic potential.

## 1. Introduction

After first being described in domestic cats (*Felis catus*) in Sweden in the 1970s, so-called ‘staggering disease’ was also recorded in Austria in the 1990s [1,2,3]. The name of the disease is a translation of the original Swedish designation ‘vingelsjuka‘ and refers to its most prominent clinical manifestation, hind-leg ataxia [4].

Clinically, the affected cats had a variety of neurological signs, e.g., hind-leg ataxia due to a spastic paresis/paralysis, inability to retract their claws, change in behavior, and in some cases hyperesthesia and seizures [1,2,4,5]. Histologically, all cases showed mild-to-severe non-purulent meningoencephalomyelitis. A viral cause was suspected, but the pathogen responsible for the disease remained unknown for decades [1,2,5].

In a recent study by Matiasek et al., rustrela virus (RusV, *Rubivirus strelense*, family *Matonaviridae*) was identified as the causative agent of ‘staggering disease’ in cats in Sweden, Germany, and Austria [6]. The same virus was also found retrospectively in tissue samples from lions which had been affected by non-purulent meningoencephalitis in the 1980s in two zoos in Germany [7].

RusV, first described by Bennett et al. in 2020, is closely related to rubella virus, which causes human measles [8]. RusV was detected in the brain tissues of a donkey (*Equus asinus*), a capybara (*Hydrochoerus hydrochaeris*), and a Bennett’s tree kangaroo (*Dendrolagus bennettianus*). These animals were all located in the same zoo in northern Germany, and all showed neurological signs before they died. Bennett et al. were also able to detect RusV in brain samples from yellow-necked field mice (*Apodemus flavicollis*), which were caught either on the zoo grounds or within a 10 km radius around the zoo. Therefore, the yellow-necked field mouse was suspected to be the reservoir host of RusV [8]. In addition, fatal RusV infections were also identified in red-necked wallabies (*Notamacropus rufogriseus*), a ring-tailed coati (*Nasua nasua*), and a Eurasian river otter (*Lutra lutra*) [9,10].

In this retrospective study, we analyzed archived formalin-fixed paraffin-embedded (FFPE) samples from 23 cats diagnosed with nonsuppurative encephalitis between 1994 and 2016 for the presence of RusV genetic material using reverse transcription real-time polymerase chain reaction (RT-qPCR) and in situ hybridization (RNAscope assay) [11]. The major aim was to find out whether RusV-associated encephalitis cases had been present throughout the entire sampling period and if the distribution area was larger than originally described [2,6].

## 2. Materials and Methods

### 2.1. Database Search

First, the database of necropsy reports of the Institute of Pathology, University of Veterinary Medicine, Vienna, including the years 1994 through 2022, was searched for the following keywords: cat + encephalitis/meningoencephalitis/meningoencephalomyelitis.

Subsequently, the archived files of the retrieved cases (accompanying letters, necropsy notes, and necropsy reports) were studied thoroughly. Our intention was to identify cases with matching clinical signs (e.g., ataxia, hind leg weakness, change in behavior, seizures) and/or histological findings (non-purulent encephalitis/meningoencephalitis/meningoencephalomyelitis of unclear etiology). Some of the accompanying letters from the 1990s already mentioned ‘staggering disease’ as the suspected diagnosis.

Based on the criteria mentioned above, we identified 23 cases, of which brain and spinal cord (if available) samples were retrieved from the archive for further analyses.

### 2.2. Selected Samples

Of these 23 cats, FFPE blocks were available from the brain tissues of all cases and from the spinal cord samples of eight cases. In one case, a freshly taken brain sample had been stored at −80 °C. All samples originated from domestic cats with ‘staggering disease’-like signs and were collected between 1994 and 2016. The majority lived in Vienna and the surrounding Lower Austria, but single cases were from the federal states of Upper Austria, Styria, and Salzburg.

### 2.3. Nucleic Acid Extraction

Of the FFPE specimens, three brain sections 10 µm in thickness were transferred to 2 mL safe-lock tubes together with 1 mL xylol, briefly vortexed, and incubated at 37 °C for 20 min in a thermal shaker at 800 rpm. Thereafter, the samples were centrifuged at 16,200× *g* for 5 min, and the supernatant was carefully removed via pipetting. Then the samples were washed twice: 1 mL EtOH was added and the samples were vortexed and further solubilized in a TissueLyser II (QIAGEN, Hilden, Germany), incubated for 5 min at room temperature (RT), and centrifuged for 5 min at 16,200× *g*; the supernatant was cautiously removed, and the procedure was repeated. Finally, the tubes were opened and the pellet dried for 1 min at RT. For the lysis of the pellets, 30 µL proteinase K and 300 µL ATL tissue lysis buffer (both QIAGEN) were added, and the samples were vortexed and incubated at 55 °C in a thermal shaker at 800 rpm for 1 h. Subsequently, the samples were cooled to RT and centrifuged at 16,200× *g* for 1 min.

For one case (2003/94), a native frozen brain tissue sample was available. It was homogenized in 1 mL phosphate-buffered saline with two 2.8-mm ceramic beads (Bertin Technologies, Montigny-le-Bretonneux, France) in a TissueLyser II, frozen at −80 °C for one hour, thawed, and then centrifuged at 16,200× *g* for 1 min. A 200 µL quantity of each sample’s supernatant was used for automated total nucleic acid extraction via QIAamp Viral RNA Mini Kit in a QIAcube extraction robot (both QIAGEN), according to the manufacturer’s instructions.

### 2.4. PCR Analysis

All nucleic acid extracts were subjected to several PCR assays. In order to verify that the previous processing steps were successful, a beta-actin mRNA qPCR was performed using a previously published set of primers and probe [12]. For the detection of RusV RNA, all extracts were initially screened using the recently established RT-qPCR assay panRusV-2 [6]. To exclude Borna disease virus 1 (BoDV-1) as the causative agent of the disease, all samples were additionally screened using a RT-qPCR assay established in-house, targeting the RNA-dependent RNA polymerase (*L*) gene of BoDV-1 (Table 1). RT-qPCRs were performed with Quantabio qScript XLT 1-Step RT-qPCR ToughMix (Quantabio, Beverly, MA, USA), using primers and probes at concentrations of 0.5 µM each, 2.5 µL extract, and the following conditions: 50 °C for 15 min, 95 °C for 2 min, and 45 cycles of 95 °C for 15 sec and 60 °C for 30 s. All RT-qPCRs were performed on an Applied Biosystems 7500 Real-Time PCR system (Thermo Fisher Scientific, Waltham, MA, USA), qTOWER³ G (Analytik Jena, Jena, Germany), or Rotor-Gene Q (QIAGEN).

Subsequently, cDNA was synthesized from all RusV RT-qPCR-positive as well as questionable samples using Maxima H Minus Reverse Transcriptase (Thermo Fisher Scientific) and random primers (Promega, Madison, WI, USA) according to the manufacturer’s instructions. From this cDNA, 2.5 µL were used for the following conventional RusV PCRs. Due to the lack of amplicons generated from the first-round PCRs, two nested PCR sets were designed with partly degenerated primers, especially targeting the Austrian samples according to the sequences established by Matiasek et al. [6] as well as other sequences generated in-house (see Table 1). Primers were designed using Clone Manager 9 Professional (Scientific & Educational Software, Westminster, CO, USA). Names of second-round primers indicate their respective positions within the complete genome sequence of RusV derived from cat AUT_02/1992 (acc. no. ON641041), the original tissue extract of which also served as a positive control for all RusV PCR assays [6].

All conventional PCRs were performed using QIAGEN OneStep RT-PCR Kit (QIAGEN). The following conditions were used for the first round of PCRs: 95 °C for 15 min, 50 cycles of 94 °C for 30 s, 60 °C for 30 s, and 72 °C for 30 s, followed by a final elongation step at 72 °C for 7 min. The second round of PCRs was performed using the following settings: 95 °C for 5 min, 50 cycles of 94 °C for 30 s, 56 °C for 30 s, and 72 °C for 30 s, and a final elongation at 72 °C for 7 min. All PCR results were examined via automatic gel electrophoresis on the QIAxcel Advanced System (QIAGEN). Nested PCR products (amplicon lengths 146 and 207 bp, respectively) were subjected to Sanger sequencing using Mix2Seq kits (Eurofins Genomics, Ebersberg, Germany), aligned with other RusV sequences using Clone Manager 9 Professional, and additionally subjected to BLAST search (https://blast.ncbi.nlm.nih.gov/Blast.cgi, accessed on 17 January 2023).

### 2.5. Hematoxylin-Eosin (H.E.) Staining of the FFPE Samples

All available FFPE brain and spinal cord samples from the 23 selected cases were cut into 3 μm thin slices, mounted on glass slides, and stained with hematoxylin-eosin. The slides were microscopically examined for the presence and amount of non-purulent encephalitis, meningoencephalitis, or meningoencephalomyelitis. The samples in which perivascular inflammation was detected were graded as mild, moderate, or severe, based on the extent of inflammatory cell infiltration according to a previously described scoring scheme by Matiasek et al. [6].

### 2.6. Immunohistochemistry

For each case, one brain section (level of thalamus including hippocampus) was subjected to immunohistochemistry with the anti-BoDV-antibody Bo18 (kindly provided by Dr. Sibylle Herzog, University of Gießen, Germany). The staining procedure was performed with an automated immunostainer (Thermo Autostainer 360-2D System) using the Ultravision LP Detection System (both Thermo Fisher Scientific) as previously described [13]. The dilution of the primary antibody was 1:30,000. A BoDV-1-positive horse brain served as positive control, and replacement of the primary antibody by an irrelevant mouse monoclonal antibody (FIP 1CD7, Ingenasa, Madrid, Spain) was used as negative control.

### 2.7. In Situ Hybridization (ISH)

The probe used in this study was the RNAscope probe V-RusV-NP-O1 (cat. no. 1173021-C1) produced by Advanced Cell Diagnostics (Newark, NJ, USA) based on a consensus sequence of RusV from Austria designed in the highly conserved region of the 5′ end of the RusV genome. A probe designed for the mRNA of the housekeeping gene peptidyl-prolyl isomerase-B (*Felis catus*-PPIB; cat. no. 455011) served as a technical positive control. As negative control, a probe designed for bacterial dihydropicolinate reductase (DapB; cat. no. 310043) was used. In situ hybridization was performed manually using the RNAscope 2.5 High Definition RED Assay (Advanced Cell Diagnostics) according to the manufacturer’s instructions [11]. From each of the 23 cases, two brain sections (level of thalamus or midbrain, hippocampus, and cerebral cortex or level of cerebellum and medulla oblongata, respectively), and from four cases one section of spinal cord was deparaffinized and pre-treated with 1×Targeted Retrieval Solution and RNAscope Protease Plus Solution (Advanced Cell Diagnostics) before hybridization [11]. The tissues were then treated with a series of pre-amplifier and amplifier solutions as well as chromogen solution (Advanced Cell Diagnostics) and counterstained with Hematoxylin Gill No. 2 (Merck, Darmstadt, Germany). In each run, a brain section from an Austrian cat previously diagnosed with RusV infection was used as a positive control. The signals were scored according to a previously established scheme [6].

## 3. Results

### 3.1. PCR and Sequencing Results

**Figure 1:**
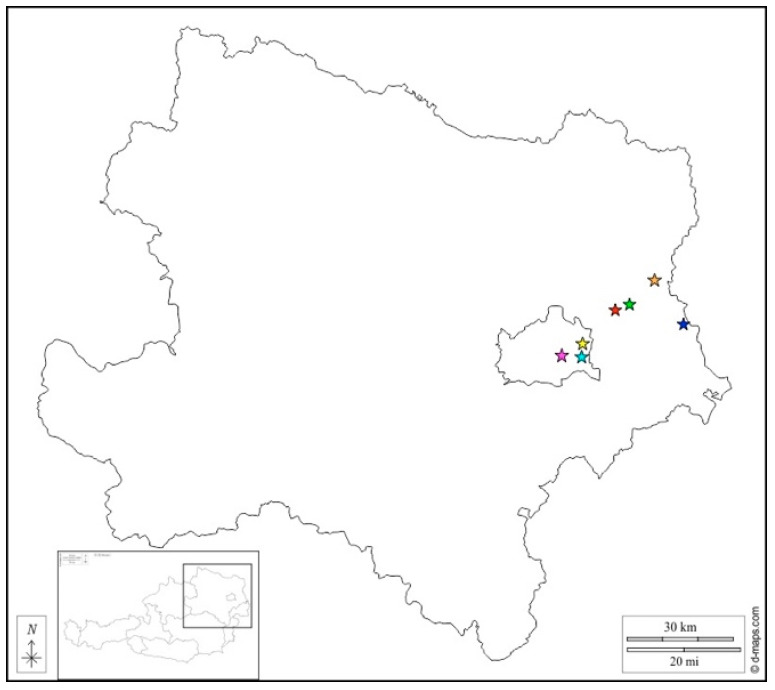
Map of Lower Austria (insert: geographical location of Lower Austria within Austria) showing the origin of the rustrela virus-infected cats (cyan: 826/94; orange: 2003/94; green: 984/96; blue: 214/97; magenta: 215/97; red: 142/98; yellow: 57/10).

In total, six of the 23 FFPE samples, as well as the one frozen sample, tested positive in a RusV RT-qPCR [Ct (cycle threshold) 34.0 to 44.0 and 26.1 to 30.9, respectively]. However, from the six FFPE samples, only two could be confirmed via conventional nested PCR, resulting in one specific sequence for each: from sample 214-97, a 144 bp-long sequence could be established from PCR 203Fn/349Rn and from sample 142-98, a 189 bp-long sequence was generated from PCR 5242Fn/5449Rn. BLAST search revealed a relationship only to other RusV sequences and showed for the first sequence 93.9% identity to AUT_09 and for the second 96.5% identity to AUT_06 (both from 1993 and first described by Matiasek et al. [6]). In addition, from the native brain tissue sample, sequences could be established from both nested PCRs, resulting in a 160 bp-long sequence from PCR 203Fn/349Rn and a 180 bp-long sequence from PCR 5242Fn/5449Rn. BLAST search revealed the closest relationship to AUT_06 (97.4–98.3%), and alignment of all generated sequences showed several nucleotide differences between all of them as well as the positive control, thereby eliminating the possibility of laboratory contamination. Furthermore, all samples tested negative in the BoDV-1 RT-qPCR. All cats diagnosed with RusV infection originated from the same geographic region of eastern Austria in which all previous cases of ‘staggering disease’ had occurred (Figure 1).

### 3.2. Histological Analysis and Immunohistochemistry

**Figure 2:**
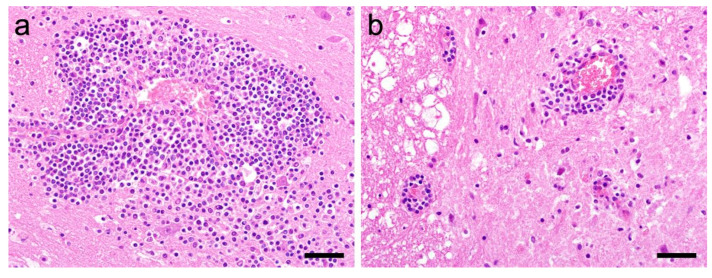
Histological features of rustrela virus-associated encephalomyelitis in cats. Large polio-predominant lymphohistiocytic perivascular cuffs in cerebral cortex (**a**) and spinal cord (**b**). Occasionally, inflammatory cells have migrated into the adjacent neuropil (**a**). Hematoxylin-eosin staining; case 984/96; bars = 40 µm.

In all 23 examined samples, non-purulent perivascular inflammation could be detected (Figure 2). The histological features (perivascular lymphohistiocytic cell infiltrates) as well as the distribution pattern (brain stem, hippocampus formation, neocortex) were consistent with the recently published study by Matiasek et al. [6]. The severity score of inflammation is shown in Table 2. In none of the cases was BoDV antigen detected via immunohistochemistry.

### 3.3. In Situ Hybridization (ISH)

We were able to detect a RusV-specific signal in seven of the 23 samples, while 16 samples were ISH-negative. All six RT-qPCR-positive samples tested positive via ISH as well (Figure S1). Interestingly, one RT-qPCR-negative sample from 1994 showed a positive ISH reaction (case 826/94).

**Figure 3:**
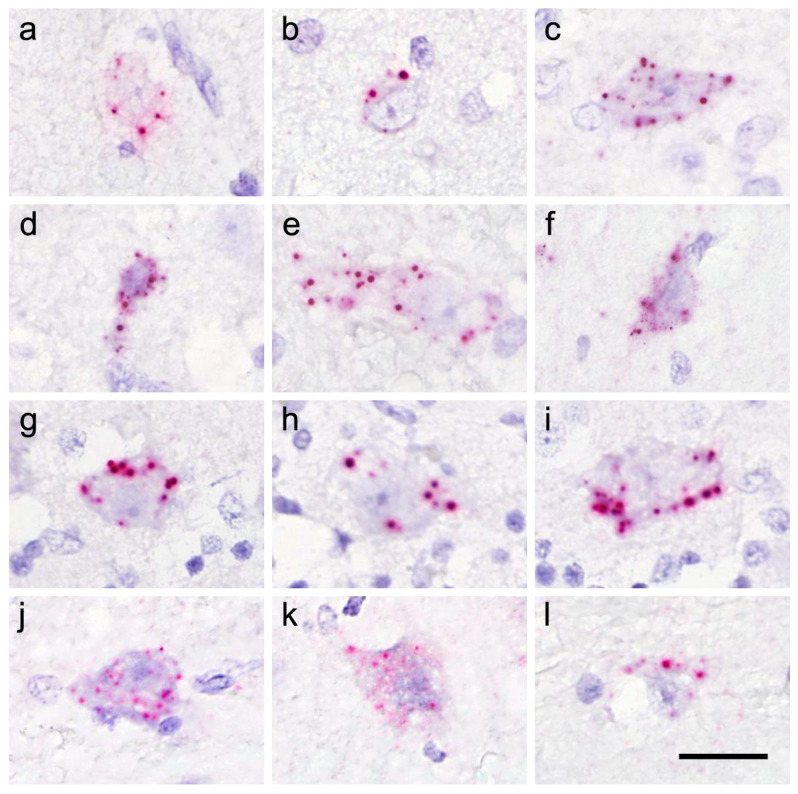
Detection of rustrela virus RNA via RNAscope in situ hybridization. Examples of staining patterns in the cytoplasm of neurons; cerebral cortex: case 57/10 (**a**,**b**), case 214/97 (**c**–**e**) and case 2003/94 (**f**); Purkinje cells: case 2003/94 (**g**–**i**); ventral horn neurons of spinal cord: case 2003/94 (**j**–**l**); bar = 10 µm.

**Figure 4:**
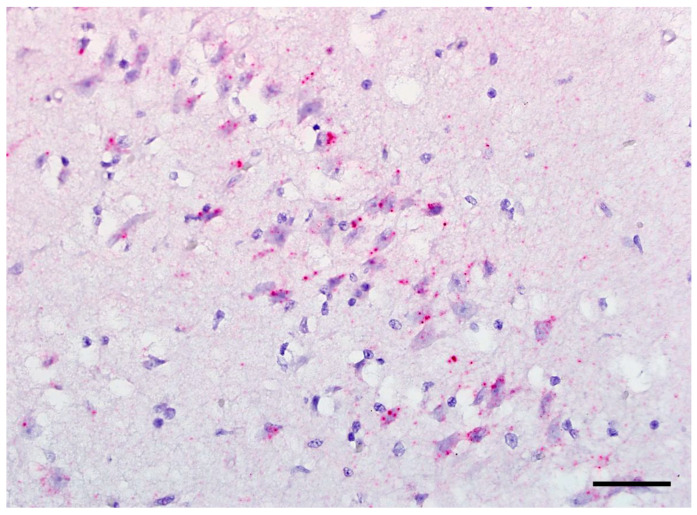
Detection of rustrela virus RNA via RNAscope in situ hybridization. Virus RNA located in the cytoplasm of numerous neurons in the pyramidal cell layer of the hippocampus; typical spherical reaction products; case 2003/94; bar = 40 µm.

The RusV signals were predominantly located in the perikarya of neurons of the cerebral cortices (mainly neocortex) (Figure 3a–f), as well as in the brain stem, the hippocampus formation (Figure 4), the cerebellum (Figure 3g–i), and in ventral horn neurons of the spinal cord (Figure 3j–l). The intensities of the signals are shown in Table 2.

**Table 2:** Geographic, demographic and clinical data of the investigated cats. Cats in which rustrela virus-associated encephalomyelitis was diagnosed are printed in bold letters.

Sample ID	Geographic Origin	Sex	Age	Month of Euthanasia/Death	Reported Anamnesis	Severity of Inflammation	RusV RT-qPCR (Ct)	RusV ISH Score
**826/94**	**1220 Wien**	**fn**	**adult**	**June 1994**	**CNS signs, blindness, deterioration despite therapy; euthanasia**	**moderate**	**-**	**1**
1942/94	3920 Hopfenleiten	mn	adult	November 1994	Aujeszky’s disease or toxicosis suspected	severe	-	-
**2003/94**	**2252 Ollersdorf**	**fn**	**3 y**	**November 1994**	**‘staggering disease’ suspected**	**severe**	**26.1–30.9 (frozen)** **38.5–44.0** **(PET)**	**3**
2250/94	1120 Wien	m	juvenile	December 1994	ataxia, weak hind extremities; euthanasia	moderate	-	-
2272/94	1040 Wien	m	1 y	December 1994	seizures with salivation, tonic–clonic convulsions for 3 days, brief improvement after administration of diazepam; animal died during seizure	mild	-	-
**984/96**	**2230 Gänserndorf**	**mn**	**adult**	**May 1996**	**‘staggering disease’ suspected**	**severe**	**35.2–40.5**	**1**
2325/96	1220 Wien	mn	5 y	December 1996	ataxia and constipation, short-term improvement with treatment; relapse with high-grade ataxia, intestinal atony, cardiovascular failure; euthanasia	severe	-	-
**214/97**	**2293 Marchegg**	**mn**	**7 y**	**January 1997**	**for 2–3 months, signs of ‘staggering disease’ which improved briefly; for 1 month, extensor cramps of all 4 extremities, uncoordinated movements**	**moderate**	**35.1–40.2**	**2**
**215/97**	**1020 Wien**	**fn**	**6 y**	**January 1997**	**for 2 months, only able to stand in a sawhorse stance, hyperesthesia to manipulation of the head**	**severe**	**38.6–43.4**	**1**
2175/97	1220 Wien	fn	10 y	October 1997	impaired balance, tonic–clonic convulsions	moderate	-	-
**142/98**	**2231 Straßhof**	**mn**	**7 y**	**January 1998**	**progressive paralysis beginning in posterior extremities, no fever; ‘staggering disease’ suspected**	**mild**	**34.0–38.2**	**2**
638/00	4722 Peuerbach	fn	2 y	March 2000	abnormal movement pattern, fever	mild	-	-
209/02	1220 Wien	mn	7 y	February 2002	epileptic seizures	severe	-	-
1114/02	2732 Willendorf	f	juvenile	June 2002	progressive central nervous signs; euthanasia	severe	-	-
2498/02	4203 Altenberg	m	6 y	December 2002	CNS signs, anorexia	mild	-	-
2026/04	1210 Wien	mn	10 y	December 2004	sudden paraparesis on the right side, loss of sensitivity on the left side of the face	moderate	-	-
1283/05	8130 Frohnleiten	fn	1 y	July 2005	salivation and vocalization, seizures, increasing weakness, unsteady gait, inappetence; euthanasia	mild	-	-
1191/06	1210 Wien	m	juvenile	July 2006	fever, anisocoria, disorientation, blindness, ataxia, tremor, inappetence; euthanasia	mild	-	-
1263/08	1210 Wien	fn	9 y	August 2008	CNS deficits, seizures, ataxia	severe	-	-
**57/10**	**1220 Wien**	**mn**	**8 y**	**January 2010**	**inappetence, apathy, exsiccosis, rhinitis**	**moderate**	**41.1–42.0**	**1**
816/13	3002 Purkersdorf	fn	6 y	July 2013	2x generalized epileptic seizures, head tremor, therapy-resistant convulsions	moderate	-	-
200/16	5721 Piesendorf	mn	juvenile	March 2016	epileptic seizures; euthanasia	moderate	-	-
490/16	3160 Traisen	mn	17 y	June 2016	acute tetraparesis, progressive blindness	severe	-	-

f: female; fn: female neutered; m: male; mn: male neutered; y: years; the RusV ISH has been scored according to Matiasek et al. [6].

## 4. Discussion

The first documented and etiologically confirmed case of RusV-associated meningoencephalitis in Austria dates back to late 1991, followed by a series of cases in 1992 and 1993 [2,3,6]. Thereafter, diseases clinically and morphologically consistent with ‘staggering disease’ have been sporadically observed but never systematically investigated.

In this retrospective study, we were able to confirm the presence of RusV in brain and spinal cord samples in seven out of 23 Austrian cats with nonsuppurative meningoencephalitis using PCR and in situ hybridization. The present survey spanned the time period between 1994 and 2016. Interestingly, six of the seven RusV-positive cases were collected during the first five years of the survey, between 1994 and 1998. Twelve years later—in 2010—a single additional positive case could be verified. After 2010, we were not able to find RusV genetic material in any of our samples. In addition, no further cases clinically suspected of being ‘staggering disease’ have been reported in Austria since. These observations raise the question of why the clinicopathologic entity ‘staggering disease’ seems to have gradually declined and finally disappeared in Austria. The situation in Sweden, the only other country with a documented endemic area with RusV infections in cats, is entirely different, because ‘staggering disease’ has never disappeared or markedly changed its incidence since the first occurrence [6].

Previous work has strongly suggested the involvement of rodent reservoir hosts in virus transmission [6,8]. Although screening of various rodent species for the presence of RusV has just started [14] and there are no data of RusV infections from Austrian rodents, it is very likely that European wood mice (*Apodemus sylvaticus*) or yellow-necked field mice (*A. flavicollis*) have also served as reservoir hosts in the endemic area in eastern Austria. These species have been hypothesized to be plausible reservoir hosts and have been found to harbor RusV without obvious clinical signs and pathological tissue lesions. Moreover, both *Apodemus* species are quite abundant in Lower Austria, albeit in geographic areas much wider than the distribution range of ‘staggering disease’ cases [15]. Based on these data, it is not very likely that the disappearance of ‘staggering disease’ in cats is related to a marked decline in the reservoir host population. Alternatively, RusV-infected individuals might have decreased in number and finally virus circulation entirely ceased. Although no histological changes could be found in brain samples of the suspected reservoir hosts in Germany [8] and Sweden [6], a connection between the presence of RusV and decreased overall fitness in the reservoir host population should be investigated. As there are no data on RusV infections of reservoir hosts in Austria, these assumptions are highly speculative to date. Further studies should be conducted to identify the responsible host and to investigate the reason for the sudden disappearance of ‘staggering disease’ in Austria.

According to current knowledge, RusV fulfills the criteria of a reservoir host-transmitted infection. The patient histories of the cats included in this study are not indicative of direct cat-to-cat transmission. Although we assume that all RusV-positive cats included in this study had outdoor access, it needs to be mentioned that we do not have solid data from all cats.

The geographic area of ‘staggering disease’ found in this study is highly consistent with the distribution pattern mentioned by Weissenböck et al. and Matiasek et al. [2,6]. This confirms the previously assumed highly sedentary nature of the involved, probably persistently infected reservoir hosts, leading to locally restricted endemic pockets in which spill-over infections in cats (and other mammals) occurred. This phenomenon is reminiscent of the geographic distribution of another infection transmitted by a small mammal reservoir host, Borna disease [13,16,17].

The majority of cases with histopathologically diagnosed nonsuppurative encephalomyelitis (69.6%) did not reveal a RusV infection. Because the selection criteria were not very stringent in order to also allow for discovery of atypical cases of ‘staggering disease’ (similar to some recent cases from Germany) [6], cats with encephalitis due to other unidentified etiologies were included. It can also not be completely excluded that samples of single cases which contained low amounts of virus were erroneously diagnosed as negative, due to the age of the samples and—with a single exception—the availability of FFPE samples only, which leads to a loss of sensitivity of PCR assays and other molecular techniques. However, it is quite evident that not all cases of nonsuppurative encephalomyelitis in cats can be attributed to RusV. RusV infections were more likely in cats originating from the defined endemic geographic area, when ataxia, paresis, or movement disorders were prominent clinical signs, and when the disease occurred during the 1990s. However, single cases (such as 57/10) presented with atypical clinical signs and outside the peak time of infections.

With this study, we were able to further substantiate RusV as the causative agent of ‘staggering disease’ and prove that sporadic cases of RusV-infected cats were common in the already described endemic area in eastern Austria during the entire decade of the 1990s. BoDV-1, which has been claimed to be the putative etiologic agent of ‘staggering disease’ in cats in Sweden, has been excluded in all cases via PCR and immunohistochemistry.

As far as we know today, RusV is able to infect a variety of different species [8] belonging to entirely unrelated orders (Carnivora, Diprotodontia, Perissodactyla, Rodentia) [8,10]. By analogy to BoDV, a possible zoonotic potential should be investigated [18,19], and it might be of interest to test non-purulent meningoencephalitides of unknown etiology in humans for the presence of RusV.

## Figures and Tables

**Table 1:** Primer/probe sets used for RusV and BoDV-1 RNA detection.

Virus	PCR Set	Primer/Probe Name	Sequence (5′ to 3′)
RusV	5F/5R (1st round)	5F	GTCGAGGAGCAGATAAGCCC
		5R	GTAACATAGTCGGGCTGGGG
	203Fn/349Rn (2nd round)	203Fn	TGCAGTCCAAGCGGTGATAC
		349Rn	CTGGGRTTCACGAGGCAATG
RusV	5113F/5681R (1st round)	5113F	CACCGAGTTCGACATGAATC
		5681R	ATGTGGCGTTCACAGCTAGA
	5242Fn/5449Rn (2nd round)	5242Fn	GTGARCCCGCGACAYTACTC
		5449Rn	GTTGCCACCCGCTTGATAGG
BoDV-1	BoDV-1 L1 RT-qPCR	202F	AGCTGCCTAATGACCTACAA
		323R	TGATCAAGAAATGACCCTTG
		236P	CAGCAGACACAGCCAAGAGCAG

## Data Availability

The data are available in electronic laboratory protocols of the participating institutions and can be made available upon request.

## Supplementary Materials

The following supporting information can be downloaded at: https://www.mdpi.com/article/10.3390/v15081621/s1, Figure S1: RNAscope in situ hybridization detects rustrela virus RNA in neurons of all cases diagnosed as positive. These neurons are either located in the cerebral cortex (826/94, 214/94, 215/94, 57/10), the Purkinje cell layer of the cerebellum (2003/94, 984/96), or in brain stem nuclei (142/98).
