# Supplementary material for: Rustrela Virus-Associated Encephalomyelitis (‘Staggering Disease’) in Cats from Eastern Austria, 1994–2016
Source: Viruses. 2023 Jul 25;15(8):1621. doi: 10.3390/v15081621 (PMC10458416; doi:10.3390/v15081621)
Supplement: Supplementary file 1 [file viruses-15-01621-s001.zip › viruses-2490997-supplementary.pdf]

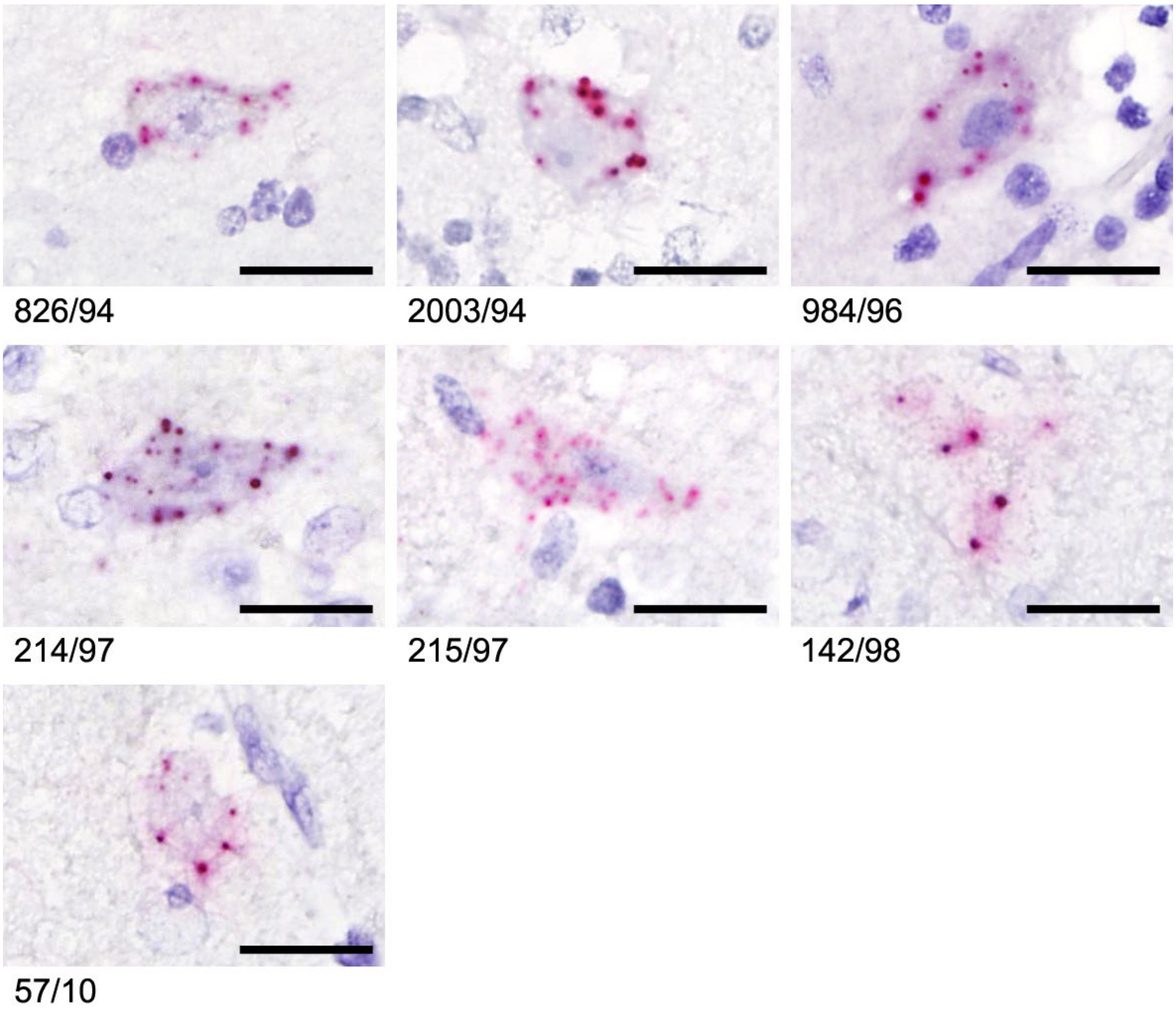

Figure S1. RNAscope in situ hybridization detects rustrela virus RNA in neurons of all cases diagnosed as positive. These neurons are either located in the cerebral cortex (826/94, 214/97, 215/97, 57/10), the Purkinje cell layer of the cerebellum (2003/94, 984/96), or in brain stem nuclei (142/98).
